# Emergence of KRAS p.G13D mutation and acquired resistance to cetuximab in colorectal cancer with vulvar metastasis: A case report

**DOI:** 10.1097/MD.0000000000018423

**Published:** 2019-12-16

**Authors:** Weiguang Qiang, Qinqin Wu, Xuefeng Ni, Chu Zhang, Jiemin Zhao

**Affiliations:** a Department of Oncology, The Third Affiliated Hospital of Soochow University, Changzhou; b Sanjing Street Community Health Service Center, Changzhou, China.

**Keywords:** acquired resistance, cetuximab, colorectal cancer, KRAS mutation, vulvar metastasis

## Abstract

**Rationale:**

Vulvar metastasis of colorectal cancer (CRC) and acquired resistance to cetuximab is a very rare phenomenon. To our knowledge, few cases have been reported in the English literatures.

**Patient concerns:**

A 55-year-old woman was diagnosed as adenocarcinoma of the rectum and the primary tumor was detected to be Kirsten-RAS (KRAS) wild type.

**Diagnoses:**

The patient was diagnosed with rectal adenocarcinoma by colonoscopy. Positron emission tomography/computed tomography (PET-CT) revealed multiple lymph node and bone metastases.

**Interventions:**

The patient received a first-line course of palliative chemotherapy with FOLFOX combined with cetuximab.

**Outcomes:**

After an initial response, acquired resistance to cetuximab occurred and vulvar metastasis was established by a second biopsy. Further molecular analysis showed that the KRAS mutation was detected in plasma samples and tumor tissues.

**Lessons:**

Vulvar metastasis from CRC is relatively rare and indicates a poor prognosis. Routine physical examinations of cutaneous and subcutaneous may facilitate early detection of metastases and timely intervention of medical technology. Moreover, combining serial tumor biopsy, liquid biopsy, and radiologic imaging could help to define mechanisms of drug resistance and to guide selection of therapeutic strategies.

## 1 Introduction

Colorectal cancer (CRC) is one of the most commonly cancer worldwide.^[1]^ The most frequent site of CRC metastasis is the liver, followed by the lung, peritoneum and bone. However, cutaneous metastases from CRC are relatively uncommon in clinical practice, with a reported frequency of about 4%.^[2]^ They usually occur in the surgical region. Invasion of vulvar skin is exceptional, which represents a major barrier to patient treatment and a poor prognosis.^[3]^ A majority of patients with metastatic colorectal cancer (mCRC) are treated with standard cytotoxic chemotherapy combined with targeted therapies, such as anti-EGFR or anti-VEGF therapies, all of which greatly extend the overall survival time of patients.^[4]^ However, after an initial response, secondary resistance to anti-EGFR therapies invariably ensues, thereby limiting the clinical benefit of this drug.^[5]^ Drug resistance resulting from alterations in Kirsten-RAS (KRAS) can be attributed not only to the selection of pre-existent KRAS mutant and amplified clones, but also to new mutations that arise as the result of continuing mutagenesis.^[6]^ Here, we present a CRC patient with a vulvar metastasis, who acquired KRAS mutation that appears to have conferred drug resistance following the administration of cetuximab and discuss it in light of the recent literature.

## 2 Case report

A 55-year-old woman who presented with hematochezia was diagnosed with adenocarcinoma of the rectum in October 2015. Imaging examination suggested multiple lymph node and bone metastases. Primary tumor tissue obtained from colonoscopy was detected to be KRAS, NRAS, and BRAF wild type. Besides, HER2 was not amplified and microsatellite stable (MSS) was identified. Then the patient received a first-line course of palliative chemotherapy with FOLFOX (Oxaliplatin 85 mg/m^2^ IV day 1, Leucovorin 400 mg/m^2^ IV day 1, 5-FU 400 mg/m^2^ IV bolus on day 1, then 1200 mg/m^2^/d×2 days IV continuous infusion, every 2 weeks) combined with cetuximab (500 mg/m^2^ IV day 1, every 2 weeks). After 4 cycles, radiologic evaluation demonstrated a partial response (PR) to treatment according to the Response Evaluation Criteria in Solid Tumors (RECIST) version 1.1, followed by maintenance therapy with cetuximab (500 mg/m^2^ IV day 1, every 2 weeks) for 4 cycles. During the period, the patient received a short course of palliative radiation (3027 cGy over 10 fractions) and a bisphosphonate (zoledronic acid) due to the cervical and thoracic spine metastasis.

**Figure 1:**
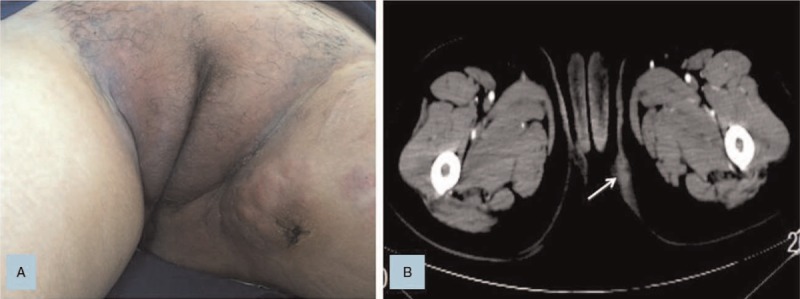
A. Nodules of the vulva were initially found. B. CT scan image for the tumor of the left vulva.

**Figure 2:**
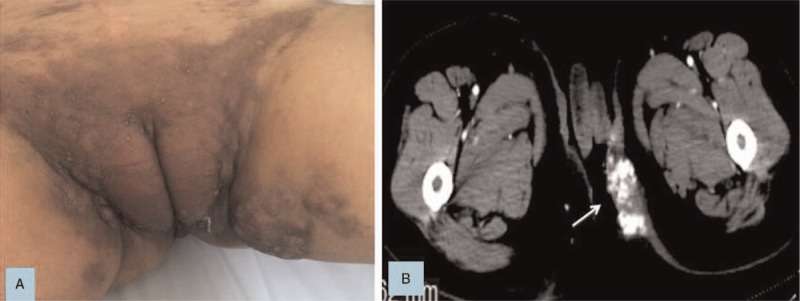
A. Vulva nodules was larger than before. B. CT scan revealed that vulvar tumor was significantly larger after multiple lines of conventional therapies and calcification was visible inside.

At the beginning of August 2016, the patient had hard nodules in the vulvar, which gradually increased and aggregated into masses (see Fig. 1). Pathological examination of skin nodules took into account metastatic cancer and was derived from the intestine. Molecular analysis showed that the KRAS p.G13D mutation was detected in plasma samples and tumor tissues. Further examination of positron emission tomography/computed tomography (PET-CT) showed widespread metastases, including lung, vertebrae, lymph nodes, and vulvar skin metastases. As the disease progressed, a second-line of chemotherapy with FOLFIRI+bevacizumab (Irinotecan 180 mg/m^2^ IV, day 1, Leucovorin 400 mg/m^2^ IV day1, 5-FU 400 mg/m^2^ IV bolus on day 1, then 1200 mg/m^2^/d×2 days IV continuous infusion, Bevacizumab 5 mg/kg IV day 1, every 2 weeks) were followed. During the couse of chemotherapy, hepatic dysfunction (grade 3) was observed according to NCI Common Terminology Criteria For Adverse Events (v 3.0) and improved after administration of the drugs. Unfortunately, the patient's vulvar lesions continued to enlarge and caused unbearable pain, forcing the patient to take painkillers (see Fig. 2). After communication with us, the patient tried to receive bevacizumab combined with paclitaxel, gemcitabine, vinorelbine, etc. successively. But the vulvar tumor continued to progress and ulcerate. Since September 2017, the apatinib (850 mg PO once daily) was taken orally and the patient found that the tumor in the vulva was slow to progress. In March 2018, the patient died of multiple organ failure in the terminal stage of the tumor and progression-free survival (PFS) was 9.8 months.

## 3 Discussion

Cutaneous metastases from internal malignancies are viewed as uncommon. In previous reports, the frequency of cutaneous metastases ranged approximately from 0.7% to 10%.^[2,7,8]^ The vulva is considered as a rare site of cutaneous metastases, and primary tumors of the cervix, ovary, and endometrium frequently metastasize to the vulua.^[9]^ In contrast, the incidence of vulvar metastases in CRC is extremely low, with only a single known study reported. In the retrospective study of 4020 patients with metastatic disease, 18 of 413 patients with mCRC had cutaneous metastases, and most of them located on the trunk, but one was on the perineum.^[2]^

In most cases, cutaneous metastases of visceral tumors indicate distant metastasis and a negative prognosis. Once diagnosed as cutaneous metastases, the time to survival drops to less than one year.^[10,11]^ However, the patient we reported had survived for 18 months after cutaneous metastases, which might benefit from the patient's very aggressive treatment. In addition, RAS and other genetic tests were not performed in early reports, and patients did not receive targeted drug therapy. Finally, many patients presented with synchronous skin metastasis at the time of diagnosis. For the reasons, their survival time was shorter.

In our case, no mutation in KRAS was detected in the primary tumor and plasma samples of rectal cancer. In contrast, in the metachronous vulvar metastasis, mutations at codon 13 (exon 2) were detected in the nodules. Mutations of the KRAS are reported to occur in about 40% of mCRC, Of which, 90% are located in exon 2 (codons 12 and 13) and 10% in others.^[12]^ A large number of clinical trials have strongly suggested that cetuximab do not provide any benefit for mCRC patients harbouring KRAS mutations.^[5,13,14]^ Indeed, KRAS mutations are by far the most common predictor of resistance to the anti-EGFR drugs cetuximab,^[15]^ and thus KRAS testing is used to select patients with KRAS wild-type mCRC for anti-EGFR treatment in clinical practice.

However, long-term clinical experience has shown that some of CRC patients with mutant KRAS can respond to cetuximab. Further studies revealed that KRAS codon 13 mutations (p.G13D) might be associated with a better outcome after cetuximab treatment than other KRAS mutations. A retrospective study including 579 chemotherapy-refractory mCRC patients treated with cetuximab indicated that patients with the KRAS p.G13D mutant tumors gained a longer overall survival (OS) and progression-free survival (PFS) in comparison with patients with tumors harboring other KRAS mutations.^[16]^ In addition, some retrospective pooled analyses suggested that cetuximab-based first-line therapy in KRAS p.G13D mutant mCRC might represent an active treatment compared with other KRAS mutations. Regarding chemotherapy, the combination with capecitabine and irinotecan was associated with a more favorable outcome.^[17–19]^ A meta-analysis of 1487 mCRC patients treated with cetuximab for any line of treatment, combining the data from ten studies, reached a conclusion similar to the above studies.^[20]^ These results were concordant with not a few in vitro studies and mouse models revealing that KRAS p.G13D mutated cell lines were more sensitive to cetuximab than other KRAS mutations cell lines.^[16,21–23]^

Surprisingly, a retrospective analysis of 110 patients was conducted to compare the therapeutic effect of cetuximab according to KRAS mutation status and showed that the patients with mutations at codon 13 of KRAS were unlikely to respond to cetuximab.^[24]^ Moreover, a phase II single-arm trial was conducted to provide prospective proof of the clinical benefit of cetuximab in KRAS p.G13D mutant mCRC patients in advanced lines of treatment. It is disappointing that no responses have been observed among 12 treated patients.^[25]^ Similar results were seen for ICECREAM study. In this randomized phase II trial, cetuximab has no activity in patients with G13D-mutated chemotherapy-refractory mCRC.^[26]^ Consistent with the above analysis, a recently published meta-analysis of eight studies found that there was not any significant difference in PFS and OS between KRAS p.G13D mutation and other KRAS mutations in terms of treatment benefit from anti-EGFR monoclonal antibody for mCRC.^[27]^

Overall, these discrepant results exist regarding KRAS p.G13D mutation, probably due to the low number of patients with such specific mutations and the heterogeneity of the sample, containing patients who were treated in multi-line chemotherapy and on different schedules. Therefore, a randomized phase III trial is warranted to further clarify the role of KRAS p.G13D.

Although a significant proportion of patients with KRAS wild type mCRC is sensitive to cetuximab treatment, acquired resistance invariably ensues in approximately 3 to 18 months.^[28]^ In our case, emergence of KRAS p.G13D mutations and acquired resistance occurred after 9 months of treatment with cetuximab. Genetic alterations of the KRAS gene (both point mutations and gene amplification) play a vital role in acquired resistance to anti-EGFR therapy in CRC. However, the question is whether these alterations are novel spontaneous mutations or the expansion of pre-existing resistant subclones during the anti-EGFR treatment. Some studies have shown a high but not complete agreement between the KRAS status in the primary tumor compared to metastatic tissue.^[29,30]^ Furthermore, the intratumoral heterogeneity of K-RAS mutations was also observed in primary tumors of mCRC.^[31]^ Diaz et al performed a study with serial samples from 28 patients followed by a mathematical modeling and concluded that KRAS mutations were most highly likely to be pre-existing in a clonal subpopulation within the WT KRAS primary tumor.^[32]^ But Misale et al conducted an in vitro experiment and declared that emergence of a cetuximab-resistant population may derive from selection of a pre-existing KRAS mutant or amplified clone or as a result of continuing mutagenesis under the pressure of cetuximab treatment.^[6]^ The mechanisms of acquired resistance to cetuximab in mCRC are far from being exhaustive due to invasiveness of the tumor biopsy and ethical issues that limit the number of feasible biopsies at progression.

Given inter- and intratumor heterogeneity genomic results from single-tumor biopsies should be interpreted with caution. By contrast, liquid biopsy approaches have the potential to detect the emergence of drug resistance during the course of treatment. In addition, KRAS variants were detectable in plasma as early as 10 months before progression was assessed by radiological methods.^[6]^ Therefore, combining serial tumor biopsy, liquid biopsy, and radiologic imaging could help to define mechanisms of drug resistance and to guide selection of therapeutic strategies. Moreover, on the basis of intratumoral heterogeneity, a phase II prospective trial was conducted and discovered that the KRAS WT CRC patients who responded and then progressed during a cetuximab-based therapy received a further line of therapy without cetuximab could restore KRAS WT clones, which could constitute the major part of the tumor mass again. Then, a rechallenge with the same cetuximab-based therapy can achieve a new clinical benefit.^[33]^ According to this study, our patient who responded initially to the cetuximab-based therapy and then progressed can try to receive, after refractory to multiple lines of chemotherapy, a further line containing the same cetuximab-based therapy gaining a clinical benefit.

Currently, management of cutaneous metastasis or recurrence of CRC is often difficult and almost exclusively pursues palliative goals. Just like our patient, visible, function impairing, unresectable cutaneous metastases always impose a burden upon the patient, associated with psychological and physiological strain. So, the majority of patients with cutaneous metastases often require systemic treatment combined with local treatment, which includes surgery and radiotherapy. Further therapeutic options include electrocoagulation and electrovaporization.^[34]^ Unfortunately, the effect is often not satisfactory. Currently, there is no evidence whether prognosis can be improved by an early detection of cutaneous metastases. But at least some cases have been offered a better local treatment result by an early detection, improving their quality of life.^[35]^ Hence routine physical examinations of cutaneous and subcutaneous may facilitate early detection of metastases and timely intervention of medical technology.

In conclusion, vulvar metastasis from CRC is relatively rare. Treatment is most often palliative due to the widespread and aggressive nature of the disease. However, there are no proposed standard therapeutic methods for mCRC of the vulva. Because of the rare frequency of this disease, large-scale prospective clinical studies are difficult to be performed. Therefore, multidisciplinary discussion is requested to bring ahead diagnostic endeavors and improved therapeutic regimen. Besides, it is certainly worth the effort of calling for self-examinations in order to attain an early detection and recognition of metastatic disease, which can dramatically change the treatment and prognosis.

## Acknowledgments

The authors are grateful to the patient for generously authorizing us to share his rare case.

## Author contributions

**Conceptualization:** Weiguang Qiang, Jiemin Zhao.

**Investigation:** Weiguang Qiang, Jiemin Zhao.

**Supervision:** Jiemin Zhao.

**Writing – original draft:** Weiguang Qiang.

**Writing – review & editing:** Weiguang Qiang, Qinqin Wu, Xuefeng Ni, Chu Zhang, Jiemin Zhao.

## References

[1] Siegel RL Miller KD Fedewa SA Colorectal cancer statistics, 2017. CA Cancer J Clin 2017;67:177–93. 28248415 10.3322/caac.21395

[2] Lookingbill DP Spangler N Helm KF Cutaneous metastases in patients with metastatic carcinoma: a retrospective study of 4020 patients. J Am Acad Dermatol 1993;29 (Pt 1):228–36. 8335743 10.1016/0190-9622(93)70173-q

[3] Akpak YK Dandin O Gun I A rare case of vulvar skin metastasis of rectal cancer after surgery. Int J Dermatol 2014;53:e337–8. 10.1111/ijd.12230 23829497

[4] Nemecek R Berkovcova J Radova L Mutational analysis of primary and metastatic colorectal cancer samples underlying the resistance to cetuximab-based therapy. Onco Targets Ther 2016;9:4695–703. 27555788 10.2147/OTT.S102891 PMC4968864

[5] Karapetis CS Khambata-Ford S Jonker DJ K-ras mutations and benefit from cetuximab in advanced colorectal cancer. N Engl J Med 2008;359:1757–65. 18946061 10.1056/NEJMoa0804385

[6] Misale S Yaeger R Hobor S Emergence of KRAS mutations and acquired resistance to anti-EGFR therapy in colorectal cancer. Nature 2012;486:532–6. 22722830 10.1038/nature11156 PMC3927413

[7] Nashan D Muller ML Braun-Falco M Cutaneous metastases of visceral tumours: a review. J Cancer Res Clin Oncol 2009;135:1–4. 18560891 10.1007/s00432-008-0432-0 PMC12160230

[8] Bansal R Patel T Sarin J Cutaneous and subcutaneous metastases from internal malignancies: an analysis of cases diagnosed by fine needle aspiration. Diagn Cytopathol 2011;39:882–7. 22081524 10.1002/dc.21485

[9] Mansouri S Glaria LA Asmae N Case of lung carcinoma revealed by vulvar metastasis associated with systemic scleroderma and literature review. Rep Pract Oncol Radiother 2013;18:182–8. 24416551 10.1016/j.rpor.2012.12.008 PMC3863135

[10] Schoenlaub P Sarraux A Grosshans E Survival after cutaneous metastasis: a study of 200 cases. Ann Dermatol Venereol 2001;128:1310–5. 11908133

[11] Braverman IM Skin manifestations of internal malignancy. Clin Geriatr Med 2002;18:1–9. v. 11913734 10.1016/s0749-0690(03)00031-4

[12] Therkildsen C Bergmann TK Henrichsen-Schnack T The predictive value of KRAS, NRAS, BRAF, PIK3CA and PTEN for anti-EGFR treatment in metastatic colorectal cancer: a systematic review and meta-analysis. Acta Oncol 2014;53:852–64. 24666267 10.3109/0284186X.2014.895036

[13] Tsoukalas N Tzovaras AA Tolia M Meta-analysis of the predictive value of KRAS mutations in treatment response using cetuximab in colorectal cancer. J BUON 2012;17:73–8. 22517696

[14] Tol J Koopman M Cats A Chemotherapy, bevacizumab, and cetuximab in metastatic colorectal cancer. N Engl J Med 2009;360:563–72. 19196673 10.1056/NEJMoa0808268

[15] Allegra CJ Jessup JM Somerfield MR American Society of Clinical Oncology provisional clinical opinion: testing for KRAS gene mutations in patients with metastatic colorectal carcinoma to predict response to anti-epidermal growth factor receptor monoclonal antibody therapy. J Clin Oncol 2009;27:2091–6. 19188670 10.1200/JCO.2009.21.9170

[16] De Roock W Jonker DJ Di Nicolantonio F Association of KRAS p.G13D mutation with outcome in patients with chemotherapy-refractory metastatic colorectal cancer treated with cetuximab. JAMA 2010;304:1812–20. 20978259 10.1001/jama.2010.1535

[17] Modest DP Reinacher-Schick A Stintzing S Cetuximab-based or bevacizumab-based first-line treatment in patients with KRAS p.G13D-mutated metastatic colorectal cancer: a pooled analysis. Anticancer Drugs 2012;23:666–73. 22441566 10.1097/CAD.0b013e328352ff1d

[18] Tejpar S Celik I Schlichting M Association of KRAS G13D tumor mutations with outcome in patients with metastatic colorectal cancer treated with first-line chemotherapy with or without cetuximab. J Clin Oncol 2012;30:3570–7. 22734028 10.1200/JCO.2012.42.2592

[19] Bando H Yoshino T Yuki S Clinical outcome of Japanese metastatic colorectal cancer patients harbouring the KRAS p.G13D mutation treated with cetuximab + irinotecan. Jpn J Clin Oncol 2012;42:1146–51. 23071293 10.1093/jjco/hys160

[20] Mao C Huang YF Yang ZY KRAS p.G13D mutation and codon 12 mutations are not created equal in predicting clinical outcomes of cetuximab in metastatic colorectal cancer: a systematic review and meta-analysis. Cancer 2013;119:714–21. 22972628 10.1002/cncr.27804

[21] Guerrero S Casanova I Farre L K-ras codon 12 mutation induces higher level of resistance to apoptosis and predisposition to anchorage-independent growth than codon 13 mutation or proto-oncogene overexpression. Cancer Res 2000;60:6750–6. 11118062

[22] Messner I Cadeddu G Huckenbeck W KRAS p.G13D mutations are associated with sensitivity to anti-EGFR antibody treatment in colorectal cancer cell lines. J Cancer Res Clin Oncol 2013;139:201–9. 23015072 10.1007/s00432-012-1319-7 PMC11824176

[23] Kumar SS Price TJ Mohyieldin O KRAS G13D mutation and sensitivity to cetuximab or panitumumab in a colorectal cancer cell line model. Gastrointest Cancer Res 2014;7:23–6. 24558511 PMC3930148

[24] Gajate P Sastre J Bando I Influence of KRAS p.G13D mutation in patients with metastatic colorectal cancer treated with cetuximab. Clin Colorectal Cancer 2012;11:291–6. 22537608 10.1016/j.clcc.2012.02.003

[25] Schirripa M Loupakis F Lonardi S Phase II study of single-agent cetuximab in KRAS G13D mutant metastatic colorectal cancer. Ann Oncol 2015;26:2503. 26371285 10.1093/annonc/mdv385

[26] Segelov E Thavaneswaran S Waring PM Response to cetuximab with or without irinotecan in patients with refractory metastatic colorectal cancer harboring the KRAS G13D Mutation: Australasian Gastro-Intestinal Trials Group ICECREAM Study. J Clin Oncol 2016;34:2258–64. 27114605 10.1200/JCO.2015.65.6843

[27] Rowland A Dias MM Wiese MD Meta-analysis comparing the efficacy of anti-EGFR monoclonal antibody therapy between KRAS G13D and other KRAS mutant metastatic colorectal cancer tumours. Eur J Cancer 2016;55:122–30. 26812186 10.1016/j.ejca.2015.11.025

[28] Misale S Di Nicolantonio F Sartore-Bianchi A Resistance to anti-EGFR therapy in colorectal cancer: from heterogeneity to convergent evolution. Cancer Discov 2014;4:1269–80. 25293556 10.1158/2159-8290.CD-14-0462

[29] Artale S Sartore-Bianchi A Veronese SM Mutations of KRAS and BRAF in primary and matched metastatic sites of colorectal cancer. J Clin Oncol 2008;26:4217–9. 18757341 10.1200/JCO.2008.18.7286

[30] Knijn N Mekenkamp LJ Klomp M KRAS mutation analysis: a comparison between primary tumours and matched liver metastases in 305 colorectal cancer patients. Br J Cancer 2011;104:1020–6. 21364579 10.1038/bjc.2011.26 PMC3065268

[31] Baldus SE Schaefer KL Engers R Prevalence and heterogeneity of KRAS, BRAF, and PIK3CA mutations in primary colorectal adenocarcinomas and their corresponding metastases. Clin Cancer Res 2010;16:790–9. 20103678 10.1158/1078-0432.CCR-09-2446

[32] Diaz LA Jr Williams RT Wu J The molecular evolution of acquired resistance to targeted EGFR blockade in colorectal cancers. Nature 2012;486:537–40. 22722843 10.1038/nature11219 PMC3436069

[33] Santini D Vincenzi B Addeo R Cetuximab rechallenge in metastatic colorectal cancer patients: how to come away from acquired resistance? Ann Oncol 2012;23:2313–8. 22396447 10.1093/annonc/mdr623

[34] Gothelf A Mir LM Gehl J Electrochemotherapy: results of cancer treatment using enhanced delivery of bleomycin by electroporation. Cancer Treat Rev 2003;29:371–87. 12972356 10.1016/s0305-7372(03)00073-2

[35] Rendi MH Dhar AD Cutaneous metastasis of rectal adenocarcinoma. Dermatol Nurs 2003;15:131–2. 12751347

